# The Expression of Circulating miR-497 and Metadherin in Hepatocellular Carcinoma: Relation to the Tumor Characteristics and Patients’ Survival

**DOI:** 10.3390/medicina57090866

**Published:** 2021-08-24

**Authors:** Dina A. Ali, Nesreen M. Sabry, Ahmed M. Kabel, Rasha A. Gaber, Hwaida M. Mokhtar, Sara M. Samy, Mohamed A. Elrashidy, Samir A. Salama, Dina Abdelhai

**Affiliations:** 1 Clinical Pathology Department, Faculty of Medicine, Tanta University, Tanta 31527, Egypt; dr.dinaadam012@gmail.com (D.A.A.); dinaibraheem85@yahoo.com (D.A.); 2 Clinical Oncology Department, Faculty of Medicine, Tanta University, Tanta 31527, Egypt; nesreensabry1eg@yahoo.com; 3 Pharmacology Department, Faculty of Medicine, Tanta University, Tanta 31527, Egypt; 4 Medical Biochemistry Department, Faculty of Medicine, Tanta University, Tanta 31527, Egypt; dr_rashagaber@yahoo.com; 5 Radiodiagnosis Department, Faculty of Medicine, Tanta University, Tanta 31527, Egypt; hwaidamahmoudhm18@gmail.com; 6 Microbiology and Immunology Department, Faculty of Medicine, Tanta University, Tanta 31527, Egypt; dr.sms2006@hotmail.com; 7 Pathology Department, Faculty of Medicine, Tanta University, Tanta 31527, Egypt; mohamedrashidy@yahoo.com; 8 Division of Biochemistry, Department of Pharmacology, College of Pharmacy, Taif University, P.O. Box 11099, Taif 21944, Saudi Arabia; s.salama@tu.edu.sa

**Keywords:** hepatocellular carcinoma, miRNA-497, metadherin, prognosis

## Abstract

*Objectives*: This study aimed to evaluate the prognostic significance and relationship of miR-497 and metadherin to hepatocellular carcinoma (HCC) tumor characteristics and patients’ survival. *Methods*: This study enrolled 120 (60 HCC patients and 60 healthy) subjects. Serum miR-497 and metadherin mRNA relative expression were analyzed by real-time quantitative reverse transcription polymerase chain reaction. The overall survival (OS) of HCC patients was assessed using the Kaplan–Meier curve and log-rank test. *Results*: Serum miR-497 showed statistically significant downregulation in HCC patients compared to controls (*p* < 0.001). Serum metadherin mRNA relative expression was significantly upregulated in HCC patients compared to controls (*p* < 0.001). Both serum miR-497 and metadherin mRNA expression were significantly associated with the number of tumor foci (*p* = 0.028 and 0.001, respectively), tumor size (*p* = 0.022 and <0.001, respectively), nodal metastasis (*p* = 0.003 and 0.003, respectively), distant metastasis (*p* = 0.003 and 0.003, respectively), vascular invasion (*p* = 0.040 and <0.001, respectively), and BCLC staging (*p* = 0.043 and 0.004, respectively). The overall survival was lower in patients with low miR-497 expression (*p* = 0.046) and in patients with high metadherin expression (*p* < 0.001). *Conclusions*: The expression levels of miR-497 showed downregulation in HCC patients, but metadherin expression showed upregulation. Both markers were inversely related and closely correlated with tumor characteristics and patients’ survival.

## 1. Introduction

Hepatocellular carcinoma (HCC) is the most common type of primary liver malignancy. It is the fifth most common cancer in males, and the seventh in females worldwide. Every year, more than half a million cases are newly diagnosed [1]. In developing countries, the incidence and total mortality rates of HCC represent about 84% and 83% of those worldwide, respectively [2].

In Egypt, the incidence of HCC has risen from 4% to 7.2% within the last ten years which may be due to an increase in the risk factors such as chronic Hepatitis C Virus (HCV) and hepatitis B infections [3]. Regardless of the advancement of HCC diagnostic and therapeutic tools, the patients’ prognosis is still poor due to increased metastasis and recurrence rates. There is an essential need to discover new diagnostic and prognostic markers for better prediction of disease outcomes and improvement of patients’ survival [4].

MicroRNAs (miRNAs) are a class of small noncoding RNAs of 19–25 nucleotides [5]. They may function as tumor suppressors or oncogenes and are implicated in different cellular processes that include metabolism, immunity, differentiation, proliferation, and apoptosis [6]. MicroRNA-497 (miR-497), a highly conserved miRNA located on human chromosome 17p13.1 [7], belongs to the miR-15 family (miR-15a, miR-15b, miR-16-1/2, miR-195, miR-424 and miR-497), sharing the same 3′-UTR binding seed sequence AGCAGCA [8]. Several studies had revealed that miR-497 levels are downregulated in different types of malignancies, such as melanoma, adrenocortical, cervical, breast, colorectal, and gastric carcinoma [9,10].

Metadherin was identified in 2002 for the first time in fetal astrocytes of persons exposed to HIV-1. So, it was known as Astrocyte elevated gene-1 (AEG-1). Metadherin was proven to be lysine-rich carcinoembryonic antigen-related cell adhesion molecule (CEACAM)-1-coisolated (LYRIC) protein [11]. The metadherin gene is located on the long arm of chromosome eight; the gene spans 12 exons and 11 introns. Metadherin is a transmembrane protein which controls different pathways closely associated with cancer, such as nuclear factor-kappa B (NF-κB), Wnt/b-catenin, MAPK/ERK, PI3K/AKT, and AP-1 [12]. Metadherin mediates its oncogenic action mainly through the NF-κB signaling pathway [13]. The metadherin expression level was reported to be linked to the incidence of different types of malignancies, particularly esophageal and breast carcinoma [14,15]. Moreover, it is linked to the progression and metastasis of other types of cancer, such as gastric carcinoma and osteosarcoma [16,17].

Studies had linked miR-497 and metadherin in the process of tumorigenesis and cancer progression, such as in ovarian cancer and non-small cell lung cancer. However, the relationship between the expression of miR-497 and metadherin in HCC is still unclear [18,19]. In this study, we aimed to investigate serum expression of miR-497 and metadherin in HCC patients (as noninvasive markers) and to evaluate their relationship to tumor characteristics and patients’ survival in order to assess their prognostic significance.

## 2. Methods

### 2.1. Ethical Conduct

This cross-sectional study was carried out in the Clinical Pathology and Medical Oncology Departments, Tanta University Hospital, Egypt during the period from December 2017 to May 2018. Patients were followed up for 30 months till November 2020. The study was conducted in accordance with the Declaration of Helsinki, and the protocol was approved by the Ethics Committee of the Faculty of Medicine, Tanta University, Egypt (Approval code 34416/1 on 21 January 2017). The study protocol was approved by the institutional ethics committee following the Declaration of Helsinki and signed informed consent was obtained from all included subjects before data collection.

### 2.2. Patients’ Characteristics

#### 2.2.1. Inclusion Criteria

This study included all newly diagnosed HCC cases with the following criteria: age (35–65 years), Barcelona Clinic Liver Cancer (BCLC) stages 0 to C, and Child–Pugh classes A and/or B. Diagnosis of HCC was based on the following criteria: (1) Histopathological diagnosis of HCC patients who underwent surgical resection or a percutaneous ultrasound-guided biopsy; or (2) radiological HCC diagnosis. Dynamic magnetic resonance imaging (MRI) was performed in certain cases when triphasic CT was not conclusive, according to the guidelines of the American Association for the Study of Liver Diseases. (3) Laboratory diagnosis by measuring alpha-fetoprotein for confirmation of the radiological results.

#### 2.2.2. Exclusion Criteria

Patients with a past history of HCC ablation, mixed HCC–cholangiocellular carcinoma or other extrahepatic malignancies were excluded. Furthermore, patients with other comorbidities such as diabetes mellitus, inflammatory bowel diseases (including colitis), and neurological insult were excluded.

### 2.3. The Studied Groups

Subjects involved in this study were recruited consecutively and divided into the following groups: group I—60 patients with HCC, and group II—60 healthy subjects. Healthy subjects were recruited in the same age range as the cases and after the HCC cases were recruited, so that they could be easily matched with our HCC cases with respect to age and sex.

All patients were subjected to detailed history taking, thorough clinical examination, liver function tests, and a complete blood picture.

### 2.4. Blood Sampling

Laboratory assays were performed on blood samples taken from all participants prior to receiving any treatments. An 8 mL sample of whole blood was collected by standard venipuncture in VACUETTE^®^ blood collection tubes (Greiner Bio-One, Kremsmuenster, Austria) containing K2EDTA and clot activator/Sep. EDTA blood samples were used for the molecular study. Routine investigations were performed on all participants including a complete blood count assay on a fully automated cell counter (Erma INC, PCE 210 N, Tokyo, Japan), liver function tests on a fully automated chemistry analyzer (Konelab Prime 60i, Konelab, Vantaa, Finland), AFP on automated immunoassay analyzers (Tosoh AIA 1800 ST, Tokyo, Japan), HCV antibody using commercially available kits (ORTHOR HCV Version 3.0 ELISA Test System, Raritan, NJ 08869, USA) according to the manufacturer’s instructions, and HB surface antigen using commercially available kits (Enzygnost^®^ and HBsAg, Siemens, Munich, Germany) according to the vendor’s guide.

### 2.5. Relative Quantification of Serum miR-497 by qRT-PCR

#### 2.5.1. Total RNA Extraction

Total RNA was extracted using miRNeasy Mini kits (Cat. No. 217004; Qiagen, Hilden, Germany) according to the manufacturer’s instructions. A NanoDrop^®^ 1000 spectrophotometer (Thermo Scientific, Wilmington, DE, USA) was used for measurements of both the concentration and purity of the extracted RNA. Concentrations (ng/µL) were measured by absorbance at 260 nm while the purity was checked by ratios of absorbance at 260/280 nm and at 260/230 nm. The extracted RNA was stored at −80 °C until the next step.

#### 2.5.2. Reverse Transcription

RNA was reverse transcribed using a MiScript^®^ II RT Kit (Cat. No. 218161; Qiagen, Germany) according to the producer’s protocol. The reaction mixture was carried out on a Biometra thermal cycler (Biometra GmbH, Gttingen, Germany), then stored at −20 °C for the subsequent PCR step.

#### 2.5.3. Relative Quantification

Quantification of miR-497 mRNA expression was conducted using a miScript SYBR^®^ Green PCR Kit (200) (Cat. No. 218073; Qiagen, Germany) according to the manufacturer’s guide and the endogenous control, GAPDH, was used for data normalization and relative quantification. The reaction was performed on the Step One qRT-PCR system (Applied Biosystems, CA, USA).

The primer sequence of miR-497 was F: 5′-ACACTGTGGTTTGTACGGCA-3′ and R: 5′-CTCCCCCACCCTCGCTCTAA-3′ and for the reference gene (GAPDH): F: 5′-GAC TCA TGA CCA CAGTCCATGC-3′ and R: 5′-AGA GGC AGG GAT GATGTT CTG-3′.

miR-497 relative mRNA expression was measured on the basis of the Cycle Threshold (CT) and normalized with GAPDH expression with the comparative formula 2^−Δ^Ct where {ΔCt = Ct (miR-497) − Ct (GAPDH)}.

### 2.6. Relative Quantification of Serum Metadherin by qRT-PCR

#### 2.6.1. Total RNA Extraction

RNA was firstly isolated using the miRNeasy RNA extraction kit (Qiagen, Hilden, Germany), according to the manufacturer’s protocol. The concentration and purity of all RNA eluted samples were measured as discussed before.

#### 2.6.2. Reverse Transcription

This process was performed using a high-capacity cDNA reverse transcription kit (Thermo-scientific, Waltham, MA, USA) for conversion of the isolated RNA into cDNA, then stored at −20 °C till the subsequent PCR step.

#### 2.6.3. Relative Quantification

The metadherin mRNA expression level was quantified using Maxima SYBR Green qPCR Master Mix (2X) (Thermo-scientific, Waltham, MA, USA, catalog no: K0251). The sequences of the primers used for metadherin were as follows: F: 5′-AAGAGG AAA ACT GAG CCA TCTG-3′ and R: 5′-CGG CTA ACATCC CAC TGA TAAT-3′. The reaction was carried out in the Step One qRT-PCR system (Applied Biosystems, CA, USA). Metadherin relative mRNA expression was measured as above.

### 2.7. Therapeutic Approach and Follow-up of HCC Patients

Partial hepatectomy was performed for patients who had solitary tumors with no vascular invasion. Inoperable patients, due to their poor performance status, associated comorbidity or large tumor size, were treated with transarterial hepatic chemoembolization, or radiofrequency. Patients with distant metastasis were treated with systemic therapy, either targeted therapy (Sorafenib) or fluoropyrimidine-based chemotherapy. Radiation therapy was used as a palliative treatment for patients with bone metastases. Patients were followed up every 3 months with serum α-fetoprotein and CT scan.

### 2.8. Study Endpoint

The endpoint of this study was the overall survival.

### 2.9. Statistical Analysis of the Obtained Data

The statistical analysis of the obtained results was performed using the Statistical Package for Social Sciences (SPSS version 23). Data were shown as frequencies for categorical variables, and mean ± standard deviation (SD) or median and range for numerical variables. The Kolmogorov–Smirnov test was used to verify the normality of distribution of the variables. Differences between the numerical variables were analyzed by Student’s *t*-test for two groups and an analysis of variance (ANOVA) test for more than two groups. Mann–Whitney and Kruskal–Wallis tests were used for variables not normally distributed. The correlation between miRNA-497 and metadherin mRNA expression levels was evaluated using Pearson’s correlation coefficient. The overall survival (OS) was calculated from the date of diagnosis to the time of the last follow-up visit or death. The Kaplan–Meier test was applied to construct survival curves. To verify the significance of differences between the studied groups, the exact log-rank test was used. Cox proportional hazard regression models were applied to assess the prognostic factors of the overall survival. MiRNA-497 and metadherin mRNA expression levels were grouped into high and low, considering their medians as cut-off points. *p*-values of less than 0.05 were considered statistically significant.

## 3. Results

### 3.1. Demographic and Clinicopathological Data

This cross-sectional study was conducted on 120 subjects, 60 of them were diagnosed as with HCC (group I) and 60 of them served as a control group (group II). The HCC patients involved in this study were 36 males and 24 females with a mean age of 50.1 ± 7.7 (35–63) years; 25% of them had a positive family history of HCC and 46.7% were positive smokers. Of the HCC patients, 12 (20%) were diagnosed histologically while 48 (80%) patients were diagnosed radiologically and serologically.

The control group included 39 males and 21 females with a mean age of 49.7 ± 7.1 (35–63) years; 15% of them had a positive family history of HCC and 38.3% were positive smokers (Table 1).

Regarding laboratory investigations of HCC cases, 81.7% showed elevated aspartate transaminase (AST) (Above 42 IU/L), 85% showed a decreased platelet count (less than 150 × 10^9^/L), 66.7% showed low serum albumin (less than 35 g/L), 71.7% showed elevated total bilirubin (more than 17 µmol/L), 50% showed elevated AFP (above 200 ng/mL), 70% were positive for HCV Ab and 33.3% were positive for HBs Ag (Table 2).

Regarding the tumor characteristics of HCC patients; 13.3% had more than two foci, 38.3% showed a tumor size more than 5 cm, 13.3% presented with nodal metastasis, 13.3% had distant metastasis and, 66.7% had vascular invasion. Regarding the Child–Pugh score, 41 (68.3%) patients were class A while 19 (31.7%) patients were class B. According to the BCLC staging system, patients with 0–A were 71.7%, with B were 20%, and with C were 8.3% (Table 2).

Regarding the treatment received, 4 (6.7%) patients underwent partial hepatectomy, 13 (21.7%) patients received TACE, 3 (5.0%) patients received radiofrequency, 32 (53.3%) patients received sorafenib, 8 (13.3%) patients received chemotherapy, and 8 (13.3%) patients received radiotherapy.

### 3.2. Evaluation of Serum miR-497 Relative Expression

**Figure 1:**
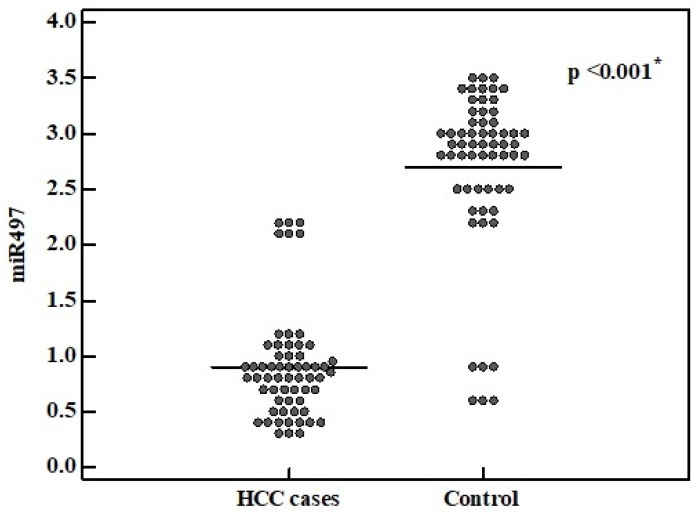
miR-497 relative expression level in HCC cases and control group.

We investigated serum expression of miR-497 in HCC cases compared to the normally matched healthy controls. We revealed that miR-497 was significantly downregulated in HCC patients compared to the controls (median value 0.8 vs. 2.9) (*p*-value < 0.001) (Figure 1).

Lower miR-497 mRNA relative expression levels were significantly related to the tumor number >2 (mean 0.6 ± 0.2, range 0.3–0.9) (*p*-value = 0.028), larger tumors >5 cm (mean 0.7 ± 0.4, range 0.3–2.2) (*p*-value = 0.022), positive nodal metastasis (mean 0.4 ± 0.1, range 0.3–0.7), distant metastasis (mean 0.4 ± 0.1, range 0.3–0.7) (*p*-value = 0.003), vascular invasion (mean 0.8 ± 0.4, range 0.3–2.2) (p-value = 0.040), stage C (mean 0.5 ± 0.2, range 0.3–0.7) (*p*-value = 0.043), and Child–Pugh class B (mean 0.8 ± 0.2, range 0.4–1.1) (Table 3).

### 3.3. Evaluation of Serum Metadherin mRNA Relative Expression

**Figure 2:**
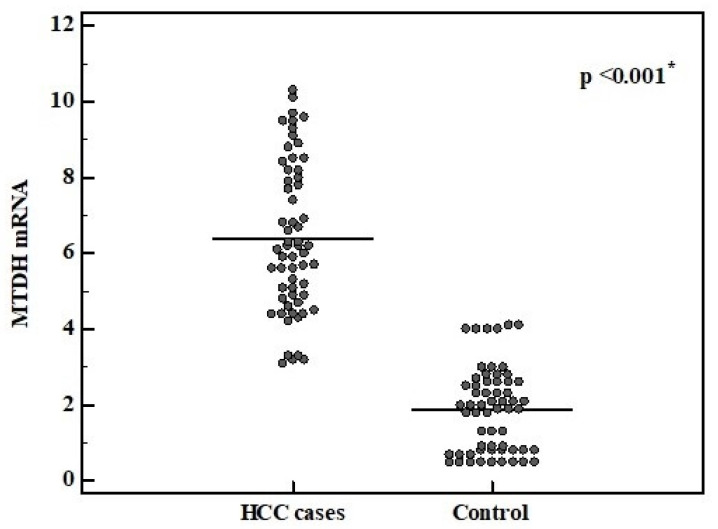
Metadherin relative expression level in HCC cases and control group.

The metadherin mRNA serum expression level was significantly upregulated in HCC cases compared to the control group (median value 6.2 vs. 2.0, *p*-value < 0.001) (Figure 2). Higher metadherin mRNA relative expression levels were significantly related to the tumor number > 2 (mean 8.6 ± 2.3, range 3.2–10.3, *p*-value = 0.001), larger tumors > 5 cm (mean 8.3 ± 1.4, range 5.2–10.3, *p*-value < 0.001), positive nodal metastasis (mean 8.3 ± 1.7, range 5.7–10.3, *p*-value = 0.003), distant metastasis (mean 8.3 ± 1.7, range 5.7–10.3, *p*-value = 0.003), vascular invasion (mean 7.4 ± 1.6, range 5.1–10.3, *p*-value < 0.001), and stage C (mean 9.1 ± 0.9, range 8.0–10.3, *p*-value = 0.004) (Table 4).

### 3.4. Relationship between miR-497 and Metadherin mRNA Expression in HCC

**Figure 3:**
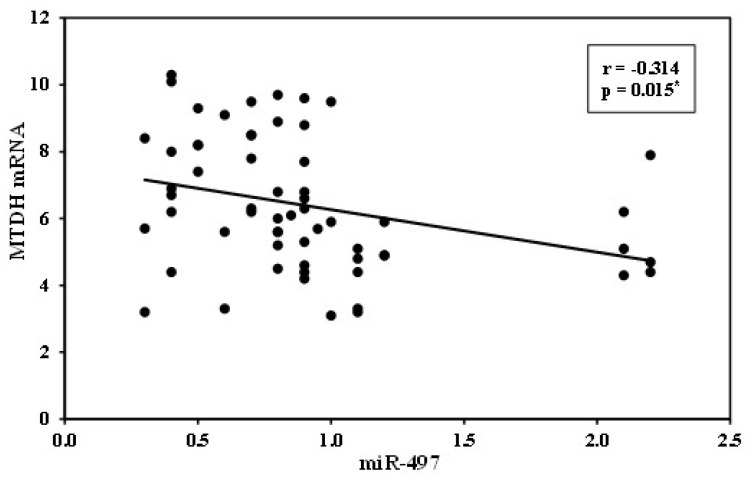
Correlation between miR-497 and metadherin expression in HCC cases (n = 60).

There was a statistically significant inverse relationship between serum miR-497 expression and serum metadherin mRNA expression (*p* 0.015) (Figure 3).

### 3.5. MiR-497 and Metadherin mRNA Expression Level and the Survival Rate

**Figure 4:**
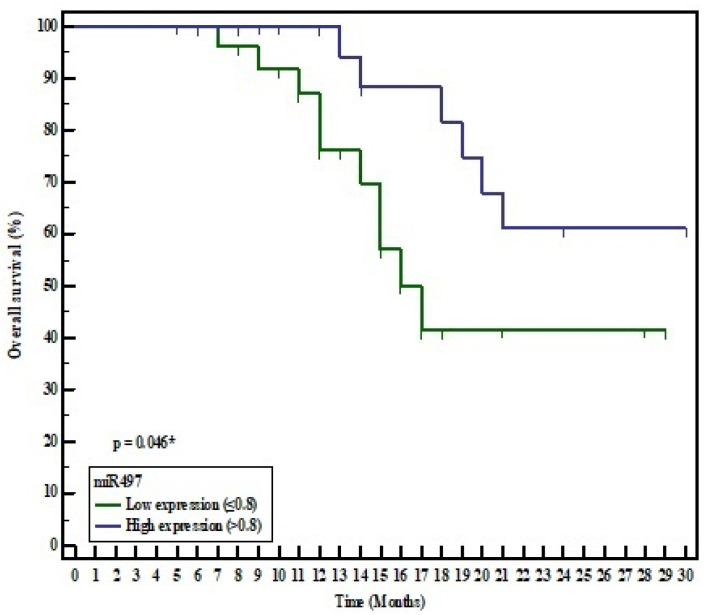
Kaplan–Meier survival curve for overall survival of HCC patients in relation to miR-497 expression.

**Figure 5:**
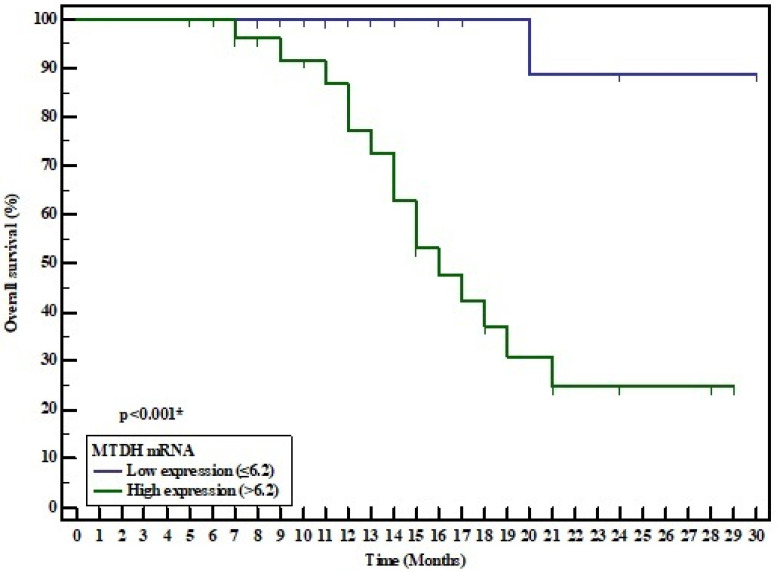
Kaplan–Meier survival curve for overall survival of HCC patients in relation to metadherin expression.

The Kaplan–Meier survival curve and log-rank test were utilized to assess the prognostic significance of both miR-497 and metadherin in HCC patients. According to the median values of their expression levels, patients were divided into high and low miR-497 expression groups (median 0.8), and high and low metadherin expression groups (median 6.2). On analyzing the overall survival (OS) time of the miR-497 groups, the survival time in the low miR-497 group was shorter than in the high miR-497 group (mean 19.91 and 25.208 months respectively). The low miR-497 group displayed a significantly poorer OS than the high miR-497 group (χ2 = 3.973, *p* = 0.046). However, the survival time in the high metadherin group was shorter than in the low metadherin group (mean 18.146 and 28.889 months respectively). The high metadherin group showed a significantly poorer OS than the low metadherin group (χ^2^ = 13.321, *p* = <0.001) (Figure 4 and Figure 5).

### 3.6. miR-497and Metadherin mRNA Relative Expression as Independent Prognostic Factors in HCC Patients

To evaluate whether miR-497 and metadherin mRNA relative expressions could serve clinically as independent prognostic factors in HCC, Cox proportional hazard regression models were performed to analyze the independent prognostic factors related to patients’ OS. Univariate analyses revealed that metadherin (HR: 2.333, *p* < 0.001), miRNA-497 (HR: 0.256, *p* = 0.031), HCV (HR: 19.931, *p* = 0.004), HBs Ag (HR: 7.243, *p* < 0.001), tumor number > 2 (HR: 6.353, *p* = 0.001), tumor size > 5 (HR: 8.410, *p* = 0.001), vascular invasion (HR: 8.752, *p* = 0.036), BCLC stage B + C (HR: 3.138, *p* = 0.026), TACE (HR: 0.125, *p* = 0.045), and sorafenib (HR: 4.399, *p* = 0.022) were significantly related to the overall survival as demonstrated in Table 5. Multivariate analyses further revealed that metadherin expression (HR: 2.783, *p* = 0.046), miRNA-497 expression (HR: 0.014, *p* = 0.038), HCV (HR: 63.436, *p* = 0.01), and tumor size > 5 (HR: 0.066, *p* = 0.045) were independently related to the overall survival in HCC cases (Table 5).

## 4. Discussion

Hepatocellular carcinoma (HCC) is the most common primary liver malignancy that represents the fifth cause of cancer-related death worldwide [20]. Although great advances in therapy have been reached, the prognosis of HCC is still low, with five-year survival rates less than 20% [21]. Most HCC cases are diagnosed late, with a high incidence of recurrence and metastasis after curative therapies that may lead to a poor prognosis. Therefore, there is an increasing need to identify novel biomarkers for better knowledge of the pathogenesis, diagnosis and prognosis of HCC [22].

Accumulating data suggest that miRNA dysregulation is frequently found in different types of tumors and plays a crucial role in the initiation and progression of cancer [23]. Until now, many studies had shown that miRNA expression is significantly different in HCC and noncancerous tissues [16]. Some miRNAs such as miR-21, miR-221, and miR-222 show upregulation in HCC [24]. These miRNAs were shown to act as oncogenes that may downregulate the expression of some tumor-suppressive genes in HCC [25]. However, miRNAs such as miR-122, miR-125b, miR-139, miR-101, and let-7 show downregulation in HCC. These miRNAs act as tumor suppressor factors by negatively regulating the different oncogenes in HCC [26].

Previous studies that focused on miRNA-497 demonstrated that its expression was downregulated in numerous tumors, and it may function as a tumor suppressor agent [27]. Moreover, its low expression had been related to poor prognosis [28]. Furthermore, the miR-497 expression level was significantly increased after primary tumor resection, suggesting that serum miR-497 may help in the evaluation of therapeutic outcomes and prediction of the prognosis of malignancy [29].

Accumulating data demonstrated that metadherin may act as an oncogene which is overexpressed in a wide range of tumors. It plays a fundamental role in carcinogenesis, angiogenesis, metastasis, and chemoresistance [30]. Metadherin was suggested to be one of the target genes of miR-497. Dysregulation of miR-497 could affect metadherin expression that results in tumorigenesis and cancer progression [31].

In the present study, our aim was to investigate serum expression of miR-497 and metadherin in HCC patients. Moreover, one of our objectives was to evaluate their expression in relation to each other, to tumor characteristics and to patients’ survival in order to assess their prognostic significance. This study was conducted on 60 HCC patients and 60 matched healthy subjects who served as a control. Our study revealed that serum miR-497 was downregulated in HCC patients compared to the normal healthy subjects. When we investigated the relationship between miR-497 expression and tumor characteristics, we noticed that miR-497 downregulation was statistically significant with the number of tumor foci, tumor size, LN metastasis, distant metastasis, vascular invasion, and BCLC staging. These results came in line with Zhang et al. [32] who found that miR-497 was downregulated in HCC tissues and its low expression was correlated with poor prognostic factors. Furthermore, Xu et al. [33] confirmed that miR-497 was significantly downregulated in HCC tumor tissues in comparison to noncancerous tissues, and postulated that miR-497 may have a role in HCC development and progression. Moreover, they showed that miR-497 downregulation was related to a large tumor size ≥ 5 cm and multiple tumor foci. On the other hand, Zhang et al. [34] discovered that the miR-497 expression level was elevated in HCC patients.

Concerning metadherin, our study revealed that the serum metadherin level was upregulated in HCC patients more than in the normal healthy subjects. This upregulation was statistically significant with the number of tumor foci, tumor size, LN metastasis, distant metastasis, vascular invasion, and BCLC staging. In a previous study performed by Jung et al. [35], it was shown that metadherin was upregulated in HCC. On analyzing the relationship between metadherin expression and the HCC clinicopathological data, they revealed that metadherin expression was strongly correlated with the size of the tumor, histological differentiation, BCLC stage, microvascular invasion and metastasis in patients with HCC. In agreement with these results, Al-sheikh et al. [36], Hu et al. [37], and Meng et al. [38] concluded that metadherin overexpression was associated with clinicopathological characters in HCC patients.

The present study revealed that decreased expression of miR-497 was correlated with overexpression of metadherin in HCC patients. This was in line with Yan et al. [39] who confirmed this inverse relationship between miR-497 and metadherin. Furthermore, they demonstrated that miR-497 suppressed the expression of metadherin by directly binding to its 3′-UTR. Moreover, restoration of metadherin could partially reverse the anti-metastasis and anti-invasion effects caused by miR-497.

To elucidate the role of miR-497 and metadherin in HCC, previous studies demonstrated that metadherin is known to be one of the target genes of miR-497. Both markers were proven to mediate their oncogenic effect via affecting the NF-κB pathway. miR-497 directly targets the regulation of inhibitor κB kinase β, which in turn enhances activation of NF-κB that increases the level of miR-497 [40]. In addition, tumor necrosis factor (TNF)-α inhibits the expression of miR-497 through negative transcriptional regulation mediated by NF-κB, indicating that there is a negative feedback loop between miR-497 and the NF-κB signaling pathway that regulates its expression [41]. Moreover, metadherin activates NF-κB directly by interacting with its p65 subunit or indirectly via degradation of NF-κB inhibitors, with subsequent upregulation of NF-κB-related gene expression that enhances cell survival, migration, and invasion, resulting in tumor progression and spread [42].

Both miR-497 and metadherin play an important role in angiogenesis which is the hallmark of cancer progression. miR-497 suppresses angiogenesis and metastasis by directly inhibiting vascular endothelial growth factor (VEGF) and metadherin, respectively [13]. Moreover, metadherin promotes the expression of angiogenic factors, such as hypoxia-inducible factor 1-α and matrix metalloproteinase-9, and enhances transformation of endothelial cells to carcinoma-associated fibroblasts [30]. Furthermore, metadherin may exert its oncogenic role via downstream mediation of the oncogenic c-Myc, and activation of the PI3K/Akt and Wnt/b-catenin signaling pathways [13].

PI3K/Akt pathway activation, which is frequently found in a variety of tumor types, affects many Akt downstream factors which are important in proliferation, apoptosis, and survival. These metadherin-regulating Akt downstream factors primarily utilize apoptosis-associated proteins such as Bcl-2, caspase-3, p27, and Forkhead box protein O1. The PI3K/Akt pathway may mediate its angiogenic effect by controlling the production of angiogenesis-associated factors such as VEGF. These results suggest that miR-497 suppresses metadherin expression, and the negative regulation of metadherin by miR-497 might contribute partially to the antimetastatic effects of miR-497 in HCC [39].

In the present study, a Kaplan–Meier analysis was carried out to evaluate OS in relation to miR-497 and metadherin expression. OS in the low miR-497 expression group was significantly lower compared to the high miR-497 expression group. However, OS in the high metadherin expression group was significantly lower compared with the low metadherin expression group. Additionally, the multivariate Cox regression analysis demonstrated that both miR-497 and metadherin could serve as independent prognostic factors in HCC.

The relationship between the survival rates and miR-497 expression in HCC was investigated by Xu et al. [33]. They showed that the HCC patients in the low miR-497 expression group exhibited worse recurrence-free survival (RFS) and overall survival (OS) than patients in the high miR-497 expression group. In 2019, a meta-analysis revealed that low expression levels of miR-497 were significantly associated with a short OS time, but no correlation was found with disease-free survival or relapse-free survival, suggesting that miR-497 is not significant as a marker for early cancer prediction, but may be used as a long-term prognostic biomarker [43].

Against our results, Zhang et al. [34] revealed that high expression of miR-497 was associated with poor survival rates in HCC patients. Zhu et al. [44] reported that the clinical outcome was consistently poorer for the high metadherin expression group than for the low metadherin expression group in the cumulative recurrence rates and in the overall survival rates. Recent studies have shown that miR-497 is upregulated in several other types of cancer [4]. Lan et al. [45] discovered that miR-497 over-expression protects tumor cells from apoptosis and enhances resistance to temozolomide in glioma. These findings indicated that miR-497 may have different roles in tumorigenesis. Moreover, miR-497 may act as an oncogene in different types of malignancies by directly targeting various downstream genes and multiple signaling pathways [46]. Furthermore, our result regarding the shorter OS in the high metadherin expression group was consistent with Ahn et al. [47], Jung et al. [35], and Xu et al. [33].

The major limitation of the present study was the relatively small sample size which may be explained by the fact that the cases were recruited from a single center. Moreover, the dynamic changes in metadherin and miR-497 expression levels in response to treatment had not been evaluated, as the effect of treatment on these markers has been reported in previous studies. Yang et al. [48] reported that the miR-497 expression level was significantly increased after surgical resection of the tumor. Abdel Ghafar et al. [49] and Lu et al. [50] also found that the plasma mRNA level of metadherin was significantly increased after chemotherapy. Therefore, further studies are recommended to evaluate the expression of miRNA-497 and metadherin in larger groups and in relation to the treatment regimen and throughout the treatment course.

## 5. Conclusions

Briefly, to the best of our knowledge, the present study was the first to investigate miR-497 and metadherin in serum of HCC patients as independent, prognostic, noninvasive genetic biomarkers. Metadherin and miR-497 may act as important key regulators in HCC progression and would be of great benefit for the identification of patients with a poor prognosis and short survival. Our study was limited by the small sample size. Furthermore, in-depth studies are essential to support our results and to elucidate the potential usefulness of miR-497 and metadherin as prognostic tools and therapeutic targets for HCC.

## Figures and Tables

**Table 1:** Demographic data of the two studied groups.

	HCC Cases (n = 60)	Control (n = 60)	Test of Significance	*p*-Value
Age (years)				
Mean ± SD.	50.1 ± 7.7	49.7 ± 7.1	t = 0.358	0.721
Median (Min.–Max.)	51.5 (35–63)	48 (35–63)		
Gender				
Male	36 (60%)	39 (65%)	χ^2^ = 0.32	0.572
Female	24 (40%)	21 (35%)		
Family history	15 (25%)	9 (15%)	χ^2^ = 1.875	0.171
Smoking	28 (46.7%)	23 (38.3%)	χ^2^ = 0.853	0.356

HCC: hepatocellular carcinoma; SD: standard deviation; Min: minimum; Max: maximum; χ^2^: Chi square test; t: Student *t*-test

**Table 2:** Distribution of the studied cases according to different parameters in hepatocellular carcinoma cases (n = 60).

	No. (%)
HCV ab	42 (70%)
HBs Ag	20 (33.3%)
Tumor number	
≤2	52 (86.7%)
>2	8 (13.3%)
Tumor size	
≤5 cm	37 (61.7%)
>5 cm	23 (38.3%)
LN metastasis	
N0	52 (86.7%)
N1	8 (13.3%)
Distant metastasis	
M0	52 (86.7%)
M1	8 (13.3%)
Vascular invasion	40 (66.7%)
Child–Pugh score	
A	41 (68.3%)
B	19 (31.7%)
BCLC staging	
0–A	43 (71.7%)
B	12 (20%)
C	5 (8.3%)
Diagnosis	
Histological	12 (20%)
Radiological/serological	48 (80%)
Treatment	
Surgical	4 (6.7%)
TACE	13 (21.7%)
Radiofrequency	3 (5%)
Targeted therapy (sorafenib)	32 (53.3%)
Chemotherapy (fluoropyrimidine)	8 (13.3%)
Radiotherapy	8 (13.3%)

HCV ab: Hepatitis C virus antibody; HBs Ag: Hepatitis B surface antigen; LN: lymph node; BCLC: Barcelona Clinic Liver Cancer.

**Table 3:** Relationship between miR-497 and different parameters in hepatocellular carcinoma cases (n = 60).

	N	miR-497		Test of Significance	*p*-Value
		Min.–Max.	Mean ± SD.		
Gender					
Male	36	0.3–1.2	0.8 ± 0.3	t = 1.884	0.070
Female	24	0.3–2.2	1.1 ± 0.7		
Family history					
No	45	0.3–2.1	0.8 ± 0.4	t = 1.407	0.178
Yes	15	0.3–2.2	1.1 ± 0.7		
Smoking					
No	15	0.3–2.2	1.0 ± 0.6	t = 0.748	0.457
Yes	45	0.3–2.2	0.9 ± 0.5		
HCV ab					
Negative	18	0.3–2.2	1 ± 0.6	t = 0.768	0.446
Positive	42	0.4–2.2	0.9 ± 0.4		
HBs Ag					
Negative	40	0.3–2.2	1.0 ± 0.5	t = 3.224 *	0.002 *
Positive	20	0.3–1.1	0.7 ± 0.2		
Tumor number					
≤2	52	0.3–2.2	1.0 ± 0.5	t = 2.250 *	0.028 *
>2	8	0.3–0.9	0.6 ± 0.2		
Tumor size					
≤5 cm	37	0.3–2.2	1.0 ± 0.5	t = 2.358 *	0.022 *
>5 cm	23	0.3–2.2	0.7 ± 0.4		
LN metastasis					
N0	52	0.3–2.2	1.0 ± 0.5	t = 3.079 *	0.003 *
N1	8	0.3–0.7	0.4 ± 0.1		
Distant metastasis					
M0	52	0.3–2.2	1.0 ± 0.5	t = 3.079 *	0.003 *
M1	8	0.3–0.7	0.4 ± 0.1		
Vascular invasion					
No	20	0.3–2.2	1.1 ± 0.5	t = 2.105 *	0.040 *
Yes	40	0.3–2.2	0.8 ± 0.4		
Child–Pugh score					
A	41	0.3–2.2	1 ± 0.6	t = 2.013 *	0.049 *
B	19	0.4–1.1	0.8 ± 0.2		
BCLC staging					
0–A	43	0.3–2.2	1.0 ± 0.5	F = 3.322 *	0.043 *
B	12	0.3–1.1	0.8 ± 0.2		
C	5	0.3–0.7	0.5 ± 0.2		

t: Student *t*-test. F: ANOVA test. *: Statistically significant at *p*-value ≤ 0.05.

**Table 4:** Relationship between metadherin and different parameters in hepatocellular carcinoma cases (n = 60).

	N	MTDH mRNA		Test of Significance	*p*-Value
		Min.–Max.	Mean ± SD.		
Gender					
Male	36	3.1–10.3	6.4 ± 2.1	t = 0.170	0.865
Female	24	3.3–9.6	6.5 ± 1.9		
Family history					
No	45	3.2–10.3	6.6 ± 1.8	t = 1.667	0.101
Yes	15	3.1–10.1	5.7 ± 2.2		
Smoking					
No	15	3.3–10.1	7.3 ± 2.3	t = 0.748	0.457
Yes	45	3.1–10.3	6.1 ± 1.8		
HCV ab					
Negative	18	3.2–8.4	5.3 ± 1.6	t = 2.887 *	0.005 *
Positive	42	3.1–10.3	6.8 ± 1.9		
HBs Ag					
Negative	40	3.1–9.6	6.0 ± 1.6	t = 2.083 *	0.047 *
Positive	20	3.2–10.3	7.2 ± 2.4		
Tumor number					
≤2	52	3.1–9.7	6.1 ± 1.7	t = 3.653 *	0.001 *
>2	8	3.2–10.3	8.6 ± 2.3		
Tumor size					
≤5 cm	37	3.1–7.8	5.2 ± 1.23	t = 8.798 *	<0.001 *
>5 cm	23	5.2–10.3	8.3 ± 1.4		
LN metastasis					
N0	52	3.1–9.7	6.1 ± 1.9	t = 3.126 *	0.003 *
N1	8	5.7–10.3	8.3 ± 1.7		
Distant metastasis					
M0	52	3.1–9.7	6.1 ± 1.9	t = 3.126 *	0.003 *
M1	8	5.7–10.3	8.3 ± 1.7		
Vascular invasion					
No	20	3.1–6.6	4.4 ± 0.9	t = 9.068 *	<0.001 *
Yes	40	5.1–10.3	7.4 ± 1.6		
Child–Pugh score					
A	41	3.2–10.1	6.2 ± 2	t = 0.908	0.367
B	19	3.1–10.3	6.7 ± 1.9		
BCLC staging					
0–A	43	3.1–10.1	6.1 ± 1.8	F = 6.067 *	0.004 *
B	12	3.2–9.6	6.3 ± 2.0		
C	5	8.0–10.3	9.1 ± 0.9		

t: Student *t*-test. F: ANOVA test. *: Statistically significant at *p* ≤ 0.05.

**Table 5:** Univariate and multivariate Cox regression analysis of prognostic markers for overall survival.

	Univariate		^#^ Multivariate	
	*p*	HR (95%CI)	*p*	HR (95%CI)
MTDH mRNA	<0.001 *	2.333 (1.509–3.607)	0.046 *	2.783 (1.019–7.601)
miR-497	0.031 *	0.256 (0.074–0.880)	0.038 *	0.014 (0.0–0.787)
HCV ab	0.004 *	19.931 (2.575–154.266)	0.010 *	63.436 (2.736–1470.744)
HBs Ag	<0.001 *	7.243 (2.488–21.082)	0.297	
Tumor number (>2)	0.001 *	6.353 (2.093–19.285)	0.657	
Tumor size (>5)	0.001 *	8.410 (2.360–29.973)	0.045 *	0.066 (0.005–0.947)
LN metastasis	0.214	2.074 (0.656–6.553)		
Distant metastasis	0.214	2.074 (0.656–6.553)		
Vascular invasion	0.036 *	8.752 (1.150–66.629)	0.972	1562.121 (0.0–4.686168)
BCLC staging (B + C)	0.026 *	3.138 (1.150–8.562)	0.093	3.771 (0.800–17.764)
Child–Pugh Class (B)	0.138	2.200 (0.777–6.227)		
Treatment				
Surgical	0.841	0.047 (0.0–42270592)		
TACE	0.045 *	0.125 (0.016–0.957)	0.968	4300.504 (0.0–1.268)
Radiofrequency	0.707	0.046 (0.0–443302.7)		
Targeted therapy (sorafenib)	0.022 *	4.399 (1.244–15.556)	0.916	0.906 (0.144–5.687)
Chemotherapy (fluoropyrimidine)	0.900	0.909 (0.206–4.007)		
Radiotherapy	0.214	2.074 (0.656–6.553)		

HR: Hazard ratio. CI: Confidence interval. ^#^: All variables with *p* < 0.05 were included in the multivariate analysis. *: Statistically significant at *p*-value ≤ 0.05.

## Data Availability

Data used and/or analyzed during this study are not available for public access because of patient privacy concerns but are available from the corresponding author upon reasonable request.
